# Cu and SnO_2_‐Modified Carbon Felt for Electroenzymatic CO_2_ Upcycling

**DOI:** 10.1002/cssc.202502316

**Published:** 2026-04-24

**Authors:** Diego Maureira, Lorena Wilson, Hilmar Guzmán, Tonia Tommasi, Debora Fino, Simelys Hernández, Carminna Ottone

**Affiliations:** ^1^ Escuela de Ingeniería Bioquímica Pontificia Universidad Católica de Valparaíso (PUCV) Valparaíso Chile; ^2^ Department of Applied Science and Technology Politecnico di Torino (PoliTO) Turin Italy

**Keywords:** bioelectrochemistry, CO_2_ conversion, formate dehydrogenase immobilization, NADH regeneration

## Abstract

This study reports scalable bioelectrodes for sustainable CO_2_‐to‐formate conversion that integrate on‐electrode cofactor regeneration with enzyme immobilization. Carbon felt (CF) supports were coated with copper (Cu) or tin oxide (SnO_2_) nanoparticles, allowing for reproducible and straightforward fabrication. Electrochemical characterization revealed that Cu‐modified electrodes (CF–NpCu) outperformed SnO_2_‐modified ones (CF‐NpSnO_2_) in NADH regeneration, achieving nearly double the faradaic efficiency (FE) toward formate and conversion yield. Coupling CF‐NpCu electrodes with affinity‐immobilized formate dehydrogenase (FDH) produced 4.4 mM formate after 5 h, a threefold increase compared to the free enzyme system. Although the free enzyme displayed higher intrinsic kinetics, immobilization positioned FDH proximal to the electrode, mitigating diffusional limitations, accelerating NADH turnover, and improving stability. The integrated system achieved a productivity of 43 µmol h^−1^ cm^−2^ and demonstrated reusability, highlighting its practical applicability. Despite moderate efficiency losses due to side reactions such as hydrogen evolution, this work establishes a scalable bioelectrode platform that effectively combines cofactor regeneration with enzymatic CO_2_ reduction, providing a promising route toward sustainable and industrially relevant electroenzymatic processes.

## Graphical abstract

Bioelectrode platform for sustainable CO_2_‐to‐formate conversion, combining electrochemical cofactor regeneration with enzyme immobilization. Cu‐modified electrodes enhanced NADH recycling compared to SnO_2_, while coupling with affinity‐immobilized formate dehydrogenase improved enzymatic turnover and stability while maintaining faradaic efficiency. The system demonstrates provides a practical route for scalable electroenzymatic CO_2_ reduction.

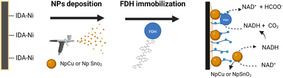

## 1 Introduction

Enzymatic electrocatalytic conversion of CO_2_ has emerged as a promising strategy for the sustainable production of value‐added chemicals, offering high selectivity and efficiency under mild conditions while providing a pathway toward scalable CO_2_ utilization [1, 2]. Among the enzymes explored for this purpose, formate dehydrogenase (FDH), which catalyzes the reversible interconversion between CO_2_ and formate, has been extensively studied as a biocatalyst for CO_2_ reduction [3, 4, 5, 6, 7, 8, 9]. Formate, in turn, can be used as a precursor for the synthesis of a wide range of chemicals, including liquid fuels and polymeric materials [10, 11].

Within the diversity of these biocatalysts, FDHs are generally classified into two groups: metal‐dependent and NAD‐dependent enzymes. Metal‐dependent FDHs, typically containing a molybdenum or tungsten active site coordinated by iron–sulfur clusters, are noted for their exceptional catalytic rates and ability to perform direct electron transfer (DET) from an electrode without exogenous cofactors [12]. However, their extreme sensitivity to oxygen and the complexity of their purification processes pose significant challenges for scalable, ambient‐pressure applications. In contrast, NAD‐dependent FDHs exhibit superior operational stability and oxygen tolerance, making them more suitable for robust bioelectrocatalytic platforms, provided the challenge of efficient cofactor regeneration is addressed [13].

Among the NAD‐dependent variants, the formate dehydrogenase from the yeast *Candida boidinii* (CbFDH) is one of the most extensively characterized and utilized biocatalysts [14]. CbFDH is particularly favored for industrial and laboratory‐scale applications due to its high selectivity for the 1,4‐NADH isomer, its well‐documented kinetic profile, and its relative stability under a range of pH and temperature conditions.

The CO_2_ conversion catalyzed by FDH utilizes NADH as a cofactor. Due to its high commercial value (≈ $2600 per mol) [15], the continuous replenishment of NADH is economically impractical. Consequently, the development of efficient NADH regeneration methods is crucial for the long‐term sustainability of this process. Most of the literature on NADH regeneration has focused on the addition of auxiliary enzyme systems or electrochemical methods [16, 17, 18]. The latter has received increasing attention due to its ability to provide a continuous supply of electrons for cofactor reduction.

Nicotinamide adenine dinucleotide (NAD) exists in two redox states: the oxidized form (NAD^+^) and the reduced form (NADH). While multiple NADH isomers exist, only the 1,4‐NADH isomer is biologically active [19]. Enzymatic reduction of NAD^+^ involves the acceptance of two electrons and one proton, yielding 1,4‐NADH. Various strategies, including chemically modified and enzyme‐mediated electrodes [18, 19, 20], have been explored for in situ NADH regeneration. While advancements have been made, many existing systems are complex, costly, or lack long‐term stability.

The integration of FDHs into electrochemical systems has enabled the development of a wide range of configurations, from simple biocathodes to complex, multifunctional platforms incorporating multiple enzymes and redox mediators [21]. A particularly impactful advancement has been the direct immobilization of FDH onto conductive electrode surfaces [16]. This approach offers clear advantages over enzyme immobilization on separate or inert supports, as it minimizes diffusional limitations by placing the biocatalyst in immediate contact with the electrode. This proximity facilitates electron transfer, significantly enhancing the overall catalytic efficiency of the system [18].

Beyond improved catalytic activity, direct immobilization provides practical benefits for system reusability and operational stability—key considerations for scalable and cost‐effective bioprocesses. Maureira et al. [22] showed that FDH immobilized on carbon felt via an affinity‐binding strategy maintained high activity over multiple CO_2_ reduction cycles, highlighting the potential for reuse without significant performance loss. Moreover, immobilization substantially enhances enzyme stability under electrochemical conditions. In contrast to free enzymes, which are prone to leaching and denaturation, immobilized dehydrogenases have demonstrated prolonged operational lifetimes. Pietricola et al. [3] reported that covalently attached enzymes on carbon felt retained electrochemical functionality and structural integrity over extended use, supporting their application in robust and durable biocathodes.

Among the materials explored for electrode construction and NADH regeneration, copper has emerged as one of the most versatile and extensively studied catalysts. Its applications range from bulk forms to nanoparticles and nanostructures with tailored crystal facets. The first report of Cu nanoparticles for electrochemical NADH regeneration was published by Song et al. (2019) [23], who developed a hybrid CO_2_ electroreduction system that coupled enzyme–cofactor conjugates with Cu‐based cofactor recycling. Since then, multiple studies have confirmed that copper not only enables high NADH regeneration yields but also achieves exceptional selectivity toward the active 1,4‐NADH isomer. Notably, Sun et al. [24] demonstrated that Cu nanowires dominated by the (111) facet achieved a 1,4‐NADH selectivity of 84.7% and a productivity of 73.5 μmol·h^−1^·cm^−2^—one of the highest reported to date. Other studies, such as García et al. [25], have also successfully implemented Cu nanoparticles in enzymatic CO_2_ conversion platforms, underscoring their versatility.

In parallel, copper nanomaterials have demonstrated outstanding performance in CO_2_ electroreduction; the performance of Cu‐based electrocatalysts is among the best achieved in transforming CO_2_ into C_1_^+^ products, particularly in forming multicarbon (C_2_^+^) products at high rates. Because Cu selectivity depends on particle size, morphology, and crystallographic orientation [26, 27, 28], we use Cu here to investigate its interaction with enzyme immobilization and cofactor regeneration.

Although tin oxide (SnO_2_) has not been previously studied as a catalyst for NADH regeneration—except in its indium‐doped form (ITO)—it has garnered increasing attention for its promising performance in CO_2_ electroreduction. SnO_2_‐based catalysts have demonstrated excellent selectivity and stability in converting CO_2_ into valuable chemicals such as formate/formic acid [29, 30]. A recent review by Song et al. [31] further emphasized the advantages of SnO_2_ in developing stable and efficient CO_2_ reduction catalysts, particularly due to its favorable electronic structure and chemical resilience. For comparison, SnO_2_ is included here as a benchmark electrocatalyst with established selectivity toward formate.

However, despite these favorable properties, a fundamental challenge remains. First, it is critical to determine whether SnO_2_ possesses the ability to catalyze NADH regeneration with sufficient activity and selectivity to compete with Cu‐based systems. Second, when CO_2_ is present in the system, it is essential to understand which electrochemical pathway is kinetically or thermodynamically favored—whether the reduction of CO_2_ or the regeneration of the nicotinamide cofactor. This competition between reaction pathways can drastically influence the system's overall performance and product distribution. Consequently, deciphering and controlling this balance is crucial for optimizing operational parameters and guiding the rational design of integrated platforms for efficient and sustainable CO_2_ valorization and enzymatic catalysis.

This study investigates the material‐specific synergy between nanoparticle chemistry and enzymatic CO_2_ reduction by comparing 3D carbon felt scaffolds modified with Cu and SnO_2_. The approach centers on the strategic interfacial confinement of *Candida boidinii* FDH to create a biocatalytic driver effect. By positioning the enzyme in the immediate proximity of the electrode, a kinetic sink is established where the rapid consumption of NADH shifts the local equilibrium, pulling the regeneration reaction forward. This integrated architecture is engineered to ensure high 1,4‐NADH selectivity even under high cathodic stress, effectively suppressing the formation of inactive dimers. Consequently, this platform provides a scalable design that bridges the gap between materials science and efficient electroenzymatic CO_2_ upcycling.

## 2 Materials and Methods

### 2.1 Materials

Recombinant *Escherichia coli* BL21 (DE3) strains transformed with pET28b‐Cb‐FDH, kanamycin resistant, and equipped with a His‐tag were used to express formate dehydrogenase (FDH) from *Candida boidinii*, following previously described protocols [22]. 3‐Glycidyloxypropyl trimethoxysilane (GPTMS), iminodiacetic acid (IDA), nickel sulfate hexahydrate (NiSO_4_·6H_2_O), toluene, acetone, potassium dihydrogen phosphate (KH_2_PO_4_), potassium hydrogen phosphate (K_2_HPO_4_), sodium bicarbonate (NaHCO_3_), sodium carbonate (Na_2_CO_3_), monosodium phosphate (H_2_NaO_4_P), disodium phosphate (Na_2_HPO_4_), nicotinamide adenine dinucleotide in reduced and oxidized forms (NADH and NAD^+^), and commercial carbon felt (3 mm thickness) were purchased from Sigma Aldrich and KWK Steek China Carbon, respectively. Copper (40–60 nm particle size) and tin oxide (<100 nm particle size) nanoparticles were purchased from Sigma–Aldrich.

### 2.2 Preparation of Modified Carbon Felt Electrodes

Carbon felt (CF) was thermally treated at 400°C in air overnight and subsequently chemically treated with nitric acid (1 M) at 100°C for 1 h [22]. The treated CF was functionalized with iminodiacetic acid (IDA) groups, and then loaded with nickel, the protocol was adapted from Pessela et al. [32]. From this point forward, the prepared support and electrode are referred to as CF‐IDA‐Ni.

The methodology used for the deposition of metal‐based particles was adapted from our previous work [33, 34]. An ink solution was prepared using Nafion (as a binder), isopropanol (as a carrier for spray deposition), and metal nanoparticles, including copper (40–60 nm) or tin oxide (particles smaller than 100 nm). A geometric area of 1 cm^2^ on the CF‐IDA‐Ni was defined for spray deposition using an airbrush with a nitrogen flow. From this point forward, the prepared support and electrode are referred to as CF‐NpCu or CF‐NpSnO_2_. To ensure a fully metallic state of copper, CF‐NpCu electrode was subjected to an electrochemical treatment using Chronopotentiometry (CP) at a constant current of −1.8 mA for 1 h using dipotassium phosphate buffer (100 mM, pH 7.0) [25]. The electrochemical reduction step was carried out in an N_2_ atmosphere at a flow rate of 30 mL min^−1^.

### 2.3 Electrode Characterization

Electrochemical measurements were performed in a two‐chamber electrochemical cell. The cell was divided into two 50 mL compartments by a 30 × 10 mm window covered with a pre‐activated Nafion 117 ion exchange membrane (DuPont). A Gamry Instruments Interface 1010E potentiostat was used to perform the electrochemical experiments. A three‐electrode configuration was employed, consisting of an Ag/AgCl (3M KCl) reference electrode (Model 012167 RE‐1B, ALS Co., Japan), a platinum wire counter electrode (CHI115, CH Instruments Inc., USA), and the prepared working electrode. 50 mL of an electrolyte solution composed of dipotassium phosphate buffer (100 mM, pH 7.0) was loaded into both the cathodic and anodic chamber. To ensure an oxygen‐free condition in the cathodic chamber, a nitrogen flow (30 NmL min^−1^) was introduced for 30 min prior to and during the experiments. Cyclic voltammetry (CV) measurements were conducted within a potential window of 0 to −1.5 V vs. Ag/AgCl at a scan rate of 30 mV s^−1^ and 5 cycles. As a control, linear sweep voltammetry (LSV) was performed using a bare CF electrode in the absence (0 mM) and presence (5 mM) of NAD^+^. The measurements were carried out between 0 and –2 V at a scan rate of 50 mV s^−1^ to assess the background electrochemical response and confirm that signals observed in subsequent experiments originate from the activity of deposited nanoparticles rather than from direct NAD^+^ oxidation at the electrode surface.

SEM images were obtained by Hitachi SU 3500 scanning electron microscope (Tokyo, Japan), equipped with a Bruker XFlash 410 M energy‐dispersive X‐ray detector with a resolution of 133 eV at the MnKα line. Secondary electron images were acquired at a magnification of 500k or higher with an EDS detector at 5 kV. The imaging conditions were working distance of 4 mm, and images were captured at magnifications of 500, 2500, 5000, 10 000, and 20 000x. FT‐IR spectra were performed to the different synthesized materials. The IR spectra were acquired with a Jasco V4600 instrument equipped with an ATR module.

### 2.4 Enzyme Activity Assay

FDH activity assay was evaluated spectrophotometrically by measuring the increase in absorbance at 340 nm, which corresponds to the release of NADH during the oxidation of formic acid at pH 7.0°C and 30°C [4]. For free FDH, dipotassium phosphate buffer 100 mM (pH 7.0), 50 mM sodium formate and 1.67 mM NAD^+^ were employed. For the immobilized FDH, 30 mg of catalyst was put in contact with 4.6 mL of 100 mM dipotassium phosphate buffer (pH 7.0), 1 mL of 300 mM formic acid and 200 µL of NAD^+^ 50 mM under vigorous stirring for 1 h at 30°C. Samples were taken every 15 min and centrifuged. The supernatant was measured at 340 nm to determine the increment of NADH in time. A unit of activity (U) was defined as the amount of enzyme that produces 1 µmol of NADH per minute under the indicated conditions.

### 2.5 NADH Regeneration

In order to evaluate NADH regeneration, Chronoamperometric (CA) analyses were performed at different potentials, −1, −1.2, −1.4 and −1.6 V vs Ag/AgCl for 30 min each on both CF‐NpCu and CF‐NpSnO_2_ electrodes. Electrochemical cells were loaded with 1 mM of NAD^+^ at the beginning of each CA and the amount of NADH formed was spectrophotometrically, 340 nm, quantified at the end of each CA. NADH regeneration efficiency was calculated according to Equation (1).

(1) $$Y_{NADH\;\text{conversion}}=\frac{\left[NADH_{end}\right]-\left[{NAD}_{\text{initial}}^{+}\right]}{\left[{NAD}_{\text{initial}}^{+}\right]}*100\%$$

Volumetric productivity was calculated following (Equation (2)):

(2) $$\text{Volumetric productivity}\;(µ\mathrm{mol}\;h^{-1}\;{\mathrm{cm}}^{-2})=\frac{C_{max}\cdot V}{t\cdot A_{electrode}}$$

Where:

$C_{max}$ = maximum formate concentration (µmol L^−1^)

*V* = reaction volume (L)

*t* = reaction time (h)

*A*_*electrode*_ = geometrical area of the electrode (cm^2^)

Faradaic efficiency (*FE*) was calculated following Equation (3).

(3) $$FE=\frac{n\cdot F\cdot mol_{P}}{\int_{0}^{t}Idt}\cdot 100\%$$

Where:

*n* = Number of electrons transferred in the reaction of interest

*F =* Faradaic constant (96485.332 C mol^−1^)

*mol*_*p*_ = Moles of product

I = Current (A)

### 2.6 Enzyme Immobilization

The enzyme immobilization of FDH onto the CF‐NpCu electrode was carried out by incubating 100 mg of CF‐NpCu with an enzymatic solution in 100 mM dipotassium phosphate buffer (pH 7.0) at 4°C, resulting in a protein loading of 12 mg g^−1^ CF and a specific activity of 4 U g^−1^. This methodology was adapted from the protocol described by Maureira et al. (2025) [22]. The immobilized support was washed and stored.

### 2.7 Formic Acid Synthesis with Electrochemical NADH Regeneration

The synthesis of formic acid with NADH regeneration was evaluated by using the CF‐NpCu electrode with 0.6 of free or immobilized FDH. The same electrochemical cell configuration described above was used. The cathodic chamber was continuously bubbled with CO_2_ at a flow rate of 30 NmL min^−1^ (standardized to 0°C and 101.325 kPa, Cole‐Parmer, model YV‐32908‐59. A solution containing 1 mM NAD^+^ prepared in 250 mM dipotassium phosphate buffer pH 8.5 was used as the electrolyte in room temperature conditions. A working potential of −1.6 V vs Ag/AgCl was applied, and samples were taken every hour to measure NADH and formic acid.

To evaluate the enzymatic utilization of the electrochemically regenerated cofactor, a stoichiometric mass balance was performed by comparing the total micromoles (µmol) of NADH produced with the total µmol of formate synthesized over a 5‐h period. The initial NADH regeneration rates (µmol h^−1^) were determined during the first hour of the CO_2_ conversion reactions to capture the steady‐state interfacial kinetics of each specific system. NADH regeneration was measured for three distinct configurations: a bare CF‐NpCu electrode (in the absence of the enzyme under N_2_ flow), using FDH in soluble form with CF‐NpCu electrode, and the immobilized bioelectrode. To isolate the specific biocatalytic contribution, the net enzymatic formate production was calculated by multiplying the electrolyte volume (20 mL) by the difference between the total measured formate concentration and the background concentration produced by the enzyme‐free CF‐NpCu electrode under identical CO_2_ flow rates (30 NmL min^−1^).

### 2.8 Compounds Analysis

The quantification of formic acid was carried out by liquid chromatography using a ThermoFisher Scientific UltiMate 3000 chromatograph equipped with a reverse phase C‐18 CORTECS column (90 Å, 2.7 µm, 4.6 X 150 mm and 250 mm) and 5 mM sulfuric acid (H_2_SO_4_) as mobile phase. The chromatographic conditions were as follows: flow rate 0.5 mL·min^−1^, injection volume 20 µL, column temperature 30°C, and UV/Visible detection at 210 nm. The concentration of NADH was determined spectrophotometrically at 340 nm (*ε* = 6.22 mM^−1^ cm^−1^) using a Jasco V730 UV–Visible spectrophotometer. A 250 mM dipotassium phosphate buffer at pH 8.5 was used as blank. All assays were performed in triplicate.

## 3 Results and Discussion

### 3.1 Electrode Characterization

**FIGURE 1:**
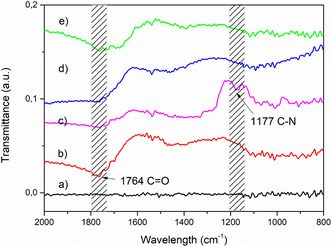
Fourier‐transform infrared (FT‐IR) spectra of the CF electrode at different treatment stages. Spectra shown are representative of duplicate measurements (*n* = 2).

To transform the naturally inert and hydrophobic carbon felt (CF) into a high‐capacity bio‐interface, a dual‐step surface modification was performed. Initially, Carbon felt (CF) was thermally treated at 400°C in air to enhance its hydrophilicity of the carbon fibers, ensuring that the 3D porous structure was accessible to aqueous media. Subsequently, nitric acid (HNO_3_) oxidation enriched the surface with hydroxyl (‐OH) and carboxyl groups, providing the necessary anchoring sites for the iminodiacetic acid (IDA) chelating agents. As confirmed by FT‐IR spectroscopy (Figure 1), the emergence of a prominent band at 1764 cm^−1^ (C=O stretching) following acid treatment indicates the successful oxygenation of the CF surface [22, 35]. The subsequent functionalization with iminodiacetic acid (IDA) groups was evidenced by the appearance of the C–N stretching band at 1177 cm^−1^ (spectrum c), confirming the successful grafting of the IDA groups required for nickel coordination and protein immobilization.

**FIGURE 2:**
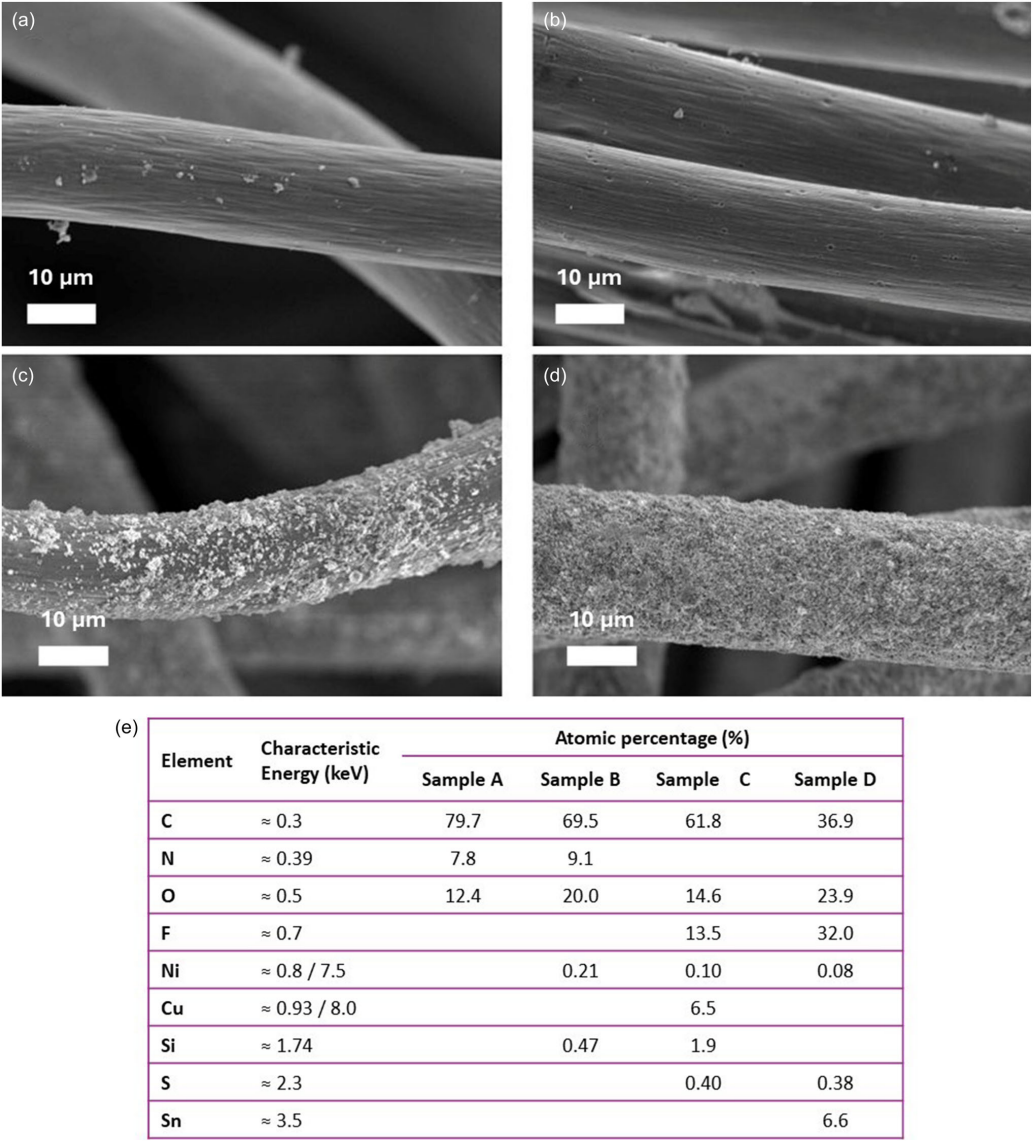
SEM images of: (a) CF control, (b) CF‐IDA‐Ni, (c) CF‐NpCu, and (d) CF‐NpSnO2. Elemental composition (atomic %) of samples determined by EDS analysis.

The morphological impact of these modifications and the subsequent nanoparticle integration were characterized by field‐emission scanning electron microscopy (FESEM) and energy‐dispersive X‐ray spectroscopy (EDS) (Figure 2). Extensive FESEM survey of the electrode surface confirmed that the morphologies shown in Figure 2 are homogeneously distributed across the 3D structure of the carbon felt. In the pristine CF (Figure 2a) and the IDA‐Ni–functionalized CF (Figure 1b), the fibers appear smooth and mostly free of surface features, with only minor irregularities. After nanoparticle deposition, the fiber morphology undergoes a marked change. In Sample C (Figure 2c), copper nanoparticles form discrete clusters on the fiber surfaces, consistent with moderate catalyst coverage. In contrast, Sample D (Figure 2d) exhibits fibers uniformly coated with a dense layer of SnO_2_ nanoparticles, reflecting higher loading and more homogeneous distribution. Energy‐dispersive X‐ray spectroscopy (EDS) analysis corroborates these observations. Sample C contains 6.5 at.% Cu and 13.5 at.% F, consistent with the presence of copper nanoparticles and a fluorine‐containing component. Sample D shows 32 at.% F and 6.6 at.% Sn, indicating substantial deposition of tin oxide (SnO_2_) nanoparticles. Carbon content decreases progressively from 79.7 at.% in Sample A (pristine CF) to 36.9 at.% in Sample D, confirming increasing nanoparticle coverage.

Overall, SEM and EDS results demonstrate successful deposition of Cu and SnO_2_ nanoparticles on CF fibers, with Sample D exhibiting the highest loading and most uniform coverage. These features are expected to enhance electrochemical activity and cofactor regeneration efficiency.

### 3.2 NADH Regeneration Performance

**FIGURE 3:**
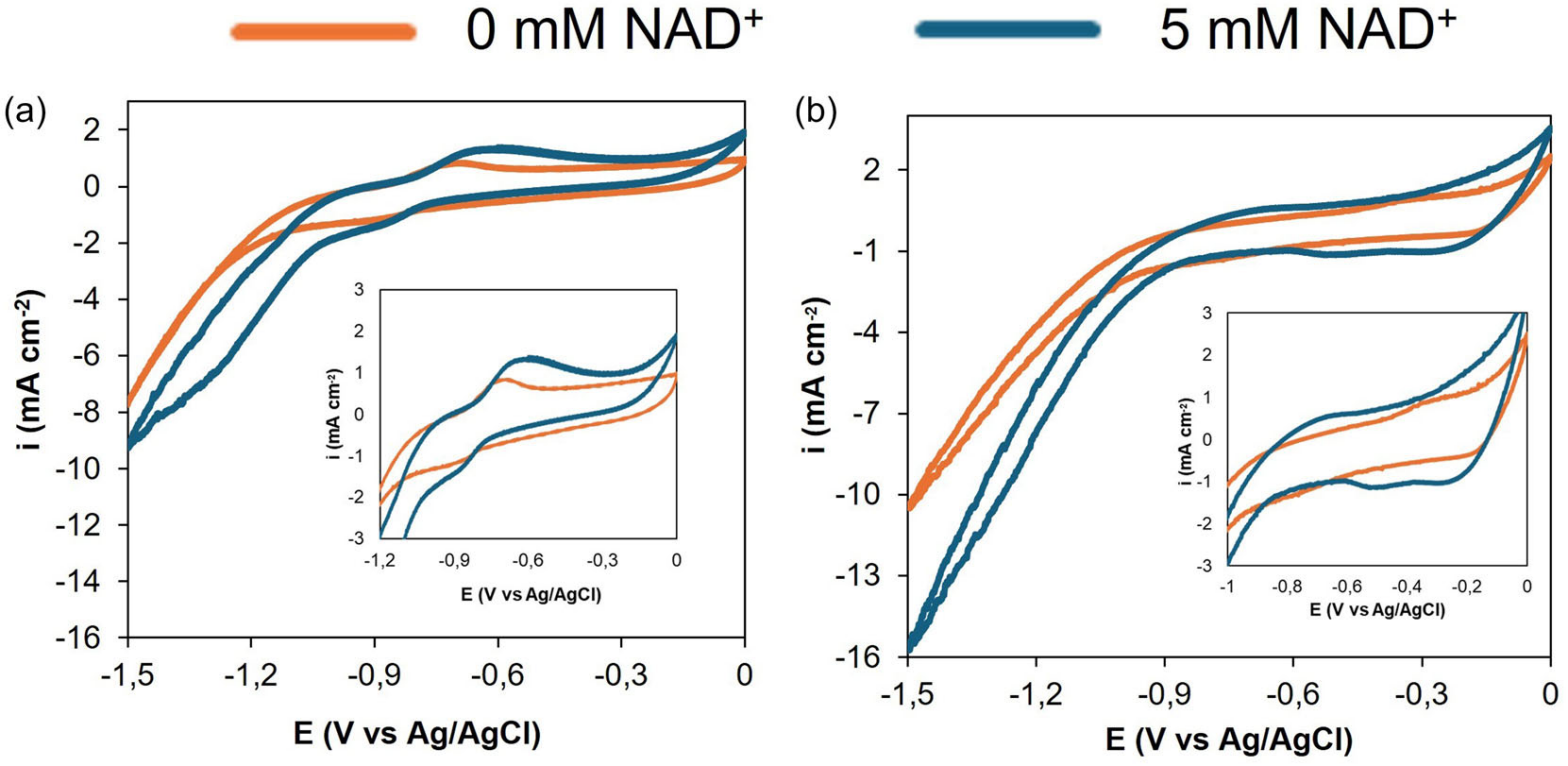
Cyclic voltammetry (CV) for the characterization of (a) CF‐NpCu and (b) CF‐NpSnO_2_ electrodes at a sweep velocity of 30 mV s^−1^. Electrolyte consisting of 100 mM dipotassium phosphate buffer (pH 7.0) with 0 mM and 5 mM of NAD^+^. All curves correspond to the 3rd stabilization cycle. The insets show a magnified view of the region from –1.2/−1.0 to 0.0 V. The inset highlights the magnified region of the reduction of NAD^+^. Curves shown are representative of triplicate measurements (*n* = 2).

Figure 3 shows the results of CV for CF–NpCu and CF–NpSnO_2_ in 100 mM dipotassium phosphate buffer (pH 7.0, 30 mV s^−1^) with 0 and 5 mM NAD^+^. Adding NAD^+^ increases the cathodic current on both electrodes, indicating NAD^+^ reduction in addition to the intrinsic electrode response (i.e., double‐layer charging, surface redox, and at more negative potentials, hydrogen evolution). For CF–NpCu (Figure 3a), NAD^+^ produces a more positive apparent onset and a steeper cathodic slope, with the NAD^+^‐associated peak centered at −0.7 V versus Ag/AgCl. Compared with the NAD^+^‐free scan, the current at the most negative potentials is higher by 9 mA cm^−2^, reflecting greater overall activity in the presence of NAD^+^. CF–NpSnO_2_ (Figure 3b) shows the same qualitative trend but activates at more negative potentials, with an onset near −0.9 V versus Ag/AgCl. Two additional cathodic shoulders appear at −0.5 and −0.2 V versus Ag/AgCl, which are plausibly associated with the reduction of surface‐adsorbed intermediates on the oxide surface. Overall, NAD^+^ reduction proceeds at milder potentials on CF–NpCu than on CF–NpSnO_2_ within the accessible window.

As a control, linear sweep voltammetry (LSV) was conducted using bare CF electrodes in the absence and presence of NAD^+^ (see Figure S1). It highlights that NAD^+^ reduction on bare CF and CF‐NpSnO_2_ (Figure 3b) occurs at potentials far more negative than the thermodynamic NAD^+^/NADH potential (–0.32 V vs. SHE at pH 7 or –0.53 V vs. Ag/AgCl) [36], with bare CF displaying reduction peaks near –1.2 V vs. Ag/AgCl, indicating sluggish kinetics and high overpotential. By contrast, CF‐NpCu shifted the reduction peak to a potential of approximately –0.7 V vs. Ag/AgCl, much closer to the theoretical value, confirming its superior catalytic activity.

**FIGURE 4:**
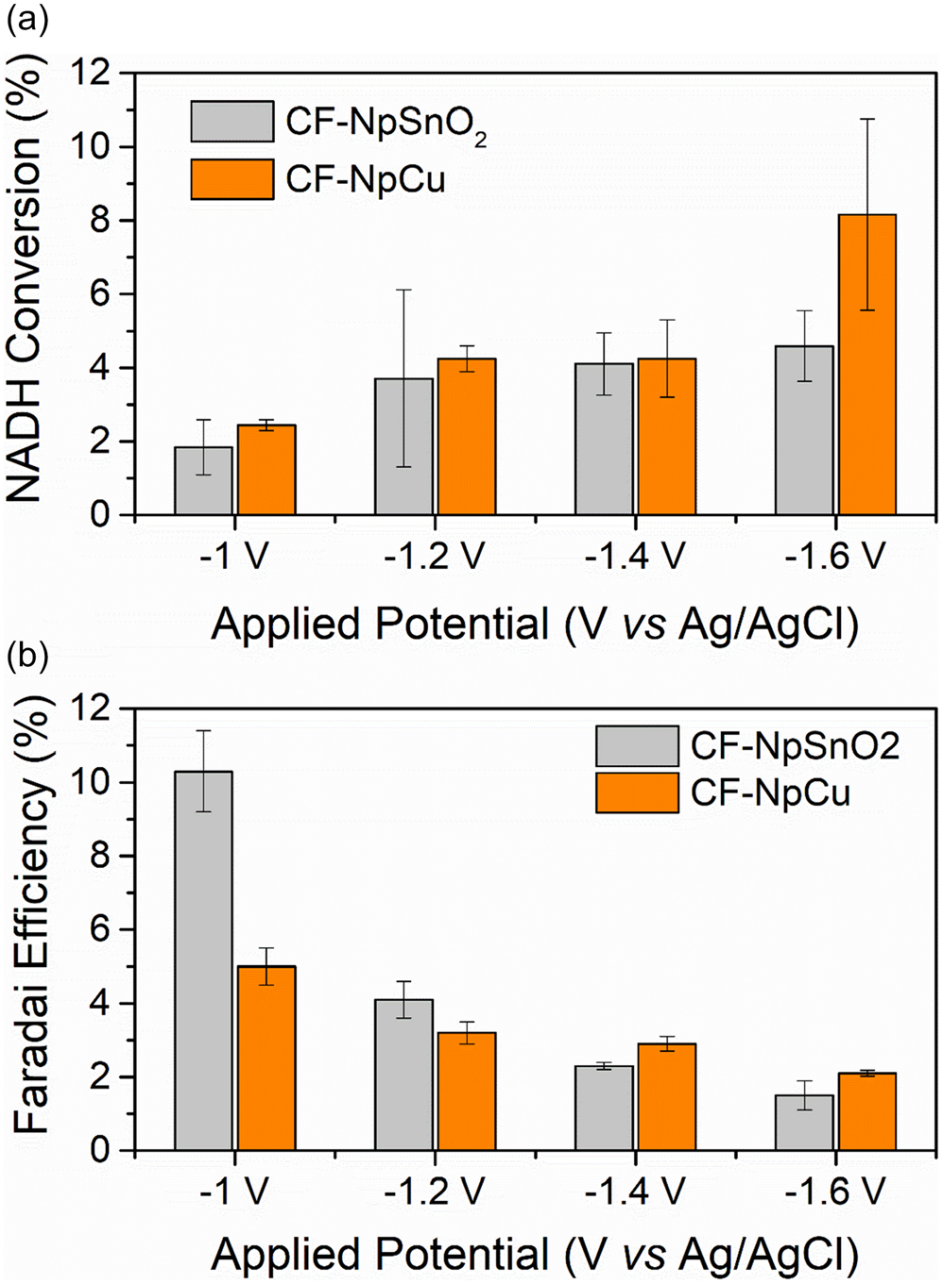
Electrochemical NADH regeneration evaluated at various applied potentials using carbon felt electrodes modified with SnO_2_ or Cu nanoparticles (CF‐NpSnO_2_ and CF‐NpCu) over a 30‐minute reaction: (a) Faradaic efficiency and (b) conversion of NAD^+^ to NADH. Data are presented as the mean of independent triplicate experiments (*n* = 3).

To identify suitable conditions for electroenzymatic NADH regeneration, controlled‐potential (chronoamperometry) experiments were performed between –1.0 and –1.6 V versus Ag/AgCl for 30 min in the presence of 1 mM NAD^+^. The selected potential window is consistent with previous studies on metallic catalyst‐based electrodes [16, 37]. The amount of regenerated NADH was quantified spectrophotometrically by monitoring the absorbance at 340 nm. Figure 4 presents the resulting regeneration efficiencies. The choice of 1 mM NAD^+^ follows Barin et al. [37], who reported inhibitory effects on FDH at elevated NADH levels. Moreover, several studies have noted that increasing the NAD^+^ concentration can diminish the regeneration yield [37, 38]. Mechanistically, NAD^+^ reduction is often described as first‐order in NAD^+^ via a two‐step sequence in which the rate‐determining step shifts with concentration: at lower NAD^+^, protonation and electro‐reduction of the NAD^+^ radical dominate, whereas at higher NAD^+^, the formation of the radical becomes rate‐limiting [39, 40]. Operating near 1 mM thus favors more efficient regeneration by promoting rapid radical turnover while ensuring sufficient electron/proton availability at the electrode interface. Practically, lower cofactor loadings also reduce consumable costs, which is advantageous for scale‐up.

The cathodic current response during NADH regeneration increases with more negative working potentials (Figure S2). Notably, the CF‐NpCu electrode exhibits significantly higher cathodic currents, with values at –1.4 and –1.6 V versus Ag/AgCl nearly double those observed for the CF‐NpSnO_2_ electrode under the same conditions. The enhanced current response further suggests that Cu provides favorable adsorption sites and efficient electron transfer pathways, thereby facilitating the reduction of NAD^+^.

The performance of the electrochemical NADH regeneration was evaluated based on the faradaic efficiency (FE) and the overall NADH conversion, as shown in Figure 4. As illustrated in Figure 4, the CF–NpCu electrode at −1.6 V versus Ag/AgCl delivered the highest NAD^+^ conversion (8.2%). A trade‐off is observed: FE decreases as the potential becomes more negative, while conversion increases. This inverse trend reflects the higher charge throughput at high overpotentials, accompanied by an increasing contribution from the hydrogen evolution reaction (HER), which diverts electrons from NAD^+^ reduction [41]. Beyond about −1.0 V vs Ag/AgCl, the conversion approaches a plateau, consistent with mass‐transfer limitations of NAD^+^/NADH at the electrode–electrolyte interface. Although FE is lower at −1.6 V, we prioritized maximizing cofactor regeneration yield for downstream coupling with FDH, accepting the selectivity penalty at this stage.

The selection of –1.6 V versus Ag/AgCl is further supported by recent systematic studies on the pH‐dependence of NAD^+^ reduction. Aamer et al. demonstrated that at alkaline conditions (pH 8.0–9.0), high cathodic potentials are required to maximize the yield of the biologically active 1,4‐NADH isomer [42]. Specifically, at pH values similar to our study (pH 8.5), potentials near –1.5 V significantly increase the percentage of active NADH compared to milder potentials, where inactive dimers or the 1,6‐isomer often dominate. Furthermore, Ali et al. [43] reported that increasing the cathodic potential on carbon‐based electrodes can dramatically improve 1,4‐NADH selectivity, reaching up to 98% at high overpotentials. While Barin et al. [37] observed NADH reduction peaks at –1.1 V using copper foam in PBS, the application of –1.6 V in our 3D carbon felt system at pH 8.5 accounts for potential drops across the porous scaffold and ensures a sufficient flux of the active cofactor to meet the catalytic demand of the enzyme.

The regeneration reaction was performed for 30 min to allow for direct comparison across potential conditions. Volumetric productivity was also assessed, with the highest value of 8.25 µmol h^−1^ cm^−2^ achieved using CF‐NpCu at –1.0 V versus Ag/AgCl (−0.88 V vs. RHE), consistent with values reported by Sun et al. (2025) [24]. Their study highlighted the critical role of electrode surface structure, cofactor concentration, and applied potential on 1,4‐NADH productivity. For example, Cu grain boundaries (111) outperformed Cu foam and Cu foil (111), with productivity increasing from 2.3 µmol h^−1^ cm^−2^ at –0.2 V versus RHE to 73.5 µmol h^−1^ cm^−2^ at –0.5 V versus RHE, and reaching 197.6 µmol h^−1^ cm^−2^ at 10 mM NAD^+^. A key goal of this work was to identify a material capable of both efficient cofactor regeneration and supporting the immobilization of FDH.

### 3.3 Formate Synthesis with Electrochemical NADH Regeneration

To construct the bioelectrode, Cb‐FDH was immobilized onto CF‐NpCu by incubation at pH 7.0 in 100 mM dipotassium phosphate buffer at 4°C. This pH condition facilitated the coordination of the His‐tag on FDH to the IDA–Ni functional groups on the electrode, resulting in a protein loading of 12 mg g^−1^ CF and a specific activity of 4 U g^−1^.

For electrocatalytic testing, the CF‐NpCu electrodes were evaluated in a two‐chamber electrochemical cell with a Nafion 117 membrane and continuous CO_2_ supply at 30 NmL min^−1^. The cathodic compartment contained 250 mM dipotassium phosphate buffer (pH 8.5) with 1 mM NAD^+^, and a potential of –1.6 V versus Ag/AgCl was applied. The choice of operating at pH 8.5 with a 1 mM NAD^+^ concentration further supports this selectivity. According to Aamer et al., more negative potentials are required at alkaline pH to favor the active monomer over inactive side products [42]. Furthermore, maintaining a 1 mM loading minimizes second‐order dimerization kinetics by reducing surface NAD^*^ radicals, favoring their protonation into active 1,4‐NADH over bimolecular coupling into dimers.

**FIGURE 5:**
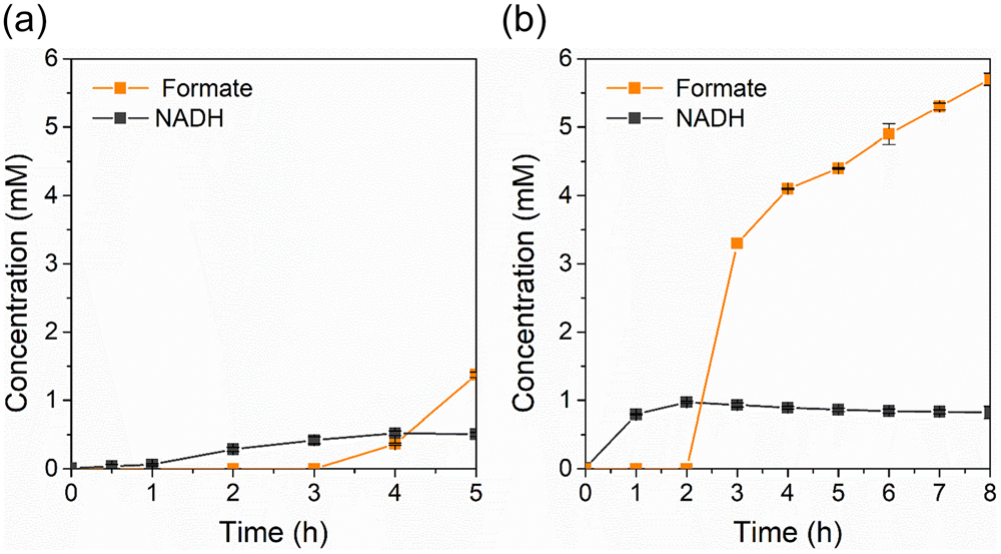
Profiles of formate and NADH production during electrochemical CO_2_ conversion at −1.6 V versus Ag/AgCl using (a) free and (b) immobilized FDH on a carbon felt electrode modified with copper nanoparticles (CF‐NpCu). The orange curves represent formate production, while the gray curves represent NADH accumulation. Experiments were conducted at room temperature with a CO_2_ flow rate of 30 NmL min^−1^ in a solution of 1 mM NAD^+^ prepared in 250 mM dipotassium phosphate buffer (pH 8.5). Data are presented as the mean of independent duplicate experiments (*n* = 2).

When using free FDH (Figure 5a), a in NADH accumulation of 0.55 mM was detectable. However, formate yields remain low, consistent with slower NADH diffusion and CO_2_‐induced enzyme deactivation. Increasing free FDH loading to 1 U caused enzyme agglomeration (Figure S3) and no detectable formate, likely due to CO_2_ sparging and mechanical shear [44, 45]. In the immobilized bioelectrode configuration, NADH formation was detectable within the first hour, while formate production exhibited a lag of ∼3 h, indicating that cofactor regeneration precedes enzymatic turnover [22, 46]. The immobilized FDH efficiently consumed the regenerated NADH, sustaining formate production with significantly enhanced stability compared with the free enzyme. After 5 h, formate concentration reached 4.4 mM, corresponding to a volumetric productivity of 43 µmol h^−1^ cm^−2^. Extended operation up to 24 h resulted in continued formate accumulation, accompanied by severe acidification (pH = 2), underscoring the importance of pH control in prolonged reactions. In contrast, the immobilized bioelectrode maintained efficient cofactor turnover, sustained formate production, and enzyme stability. Control experiments with CF‐NpCu in the absence of FDH (Figure S4) yielded only 0.6 mM formate, confirming that the enhanced conversion is due to the synergy between the electrocatalyst and immobilized FDH, mediated by the His‐tag–IDA–Ni interaction. Control experiments performed using CF‐NpCu in the absence of FDH (Figure S4) yielded a formate concentration of only 0.6 mM. This result confirms that the significantly higher formate production observed in the presence of the enzyme—both in soluble and immobilized forms—is a direct result of the enzymatic reduction of CO_2_ driven by the electrochemical regeneration of NAD^+^ to its active NADH form. When utilizing the immobilized enzyme configuration, the close proximity of the FDH to the electrode surface provides a clear kinetic advantage. This spatial arrangement facilitates the rapid consumption of regenerated NADH at the bio‐interface, effectively mitigating the mass transfer limitations inherent to the soluble enzyme system. In the latter case, NADH must diffuse from the electrode surface into the bulk solution to reach the enzyme, while NAD^+^ must migrate back to the electrode for re‐reduction. By bypassing these diffusion‐limited steps, the immobilized system ensures a higher local concentration of the cofactor at the catalytic site, thereby enhancing the overall efficiency of the formate production.

Faradaic efficiencies were similar between the bioelectrode and enzyme‐free system, indicating that immobilization did not hinder NADH regeneration. The free enzyme system achieved 3.62%, whereas the bioelectrode reached 2.49%, consistent with NADH regeneration measurements for CF‐NpCu (Figure 5a). These results suggest the rate‐limiting step is cofactor regeneration rather than enzymatic turnover, emphasizing the importance of efficient NADH regeneration. The relatively low efficiencies also indicate competition from HER or other side reactions [40], warranting further mechanistic investigation.

Thermal stability assays were performed under non‐operational conditions for both the bioelectrode and the free enzyme, incubated at 30°C and pH 7.0. Although the bioelectrode did not exhibit enhanced stability compared to the free enzyme under these non‐operational conditions (see Fig. S5), a clear improvement in stability was observed during actual operating conditions, highlighting the protective effect of immobilization under active catalytic turnover. These results indicate that immobilization not only facilitates efficient cofactor regeneration and substrate conversion but also helps maintain enzyme integrity during prolonged electrochemical operation.

The results demonstrate that the electrochemical cell, when coupled with the bioelectrode, effectively facilitated the conversion of CO_2_ to formic acid while simultaneously regenerating NADH. Significantly, the affinity‐based immobilization strategy did not compromise cofactor regeneration, maintaining efficient NADH turnover. However, the system still requires further optimization to identify the operating potential that minimizes undesired side reactions, to select a pH that balances cofactor regeneration with CO_2_ conversion, and to implement a pH control system capable of maintaining a stable environment over time, thereby preserving enzyme stability. This dual functionality highlights the efficiency of the system in both product formation and cofactor regeneration, underscoring its potential for sustainable and integrated catalytic applications.

**FIGURE 6:**
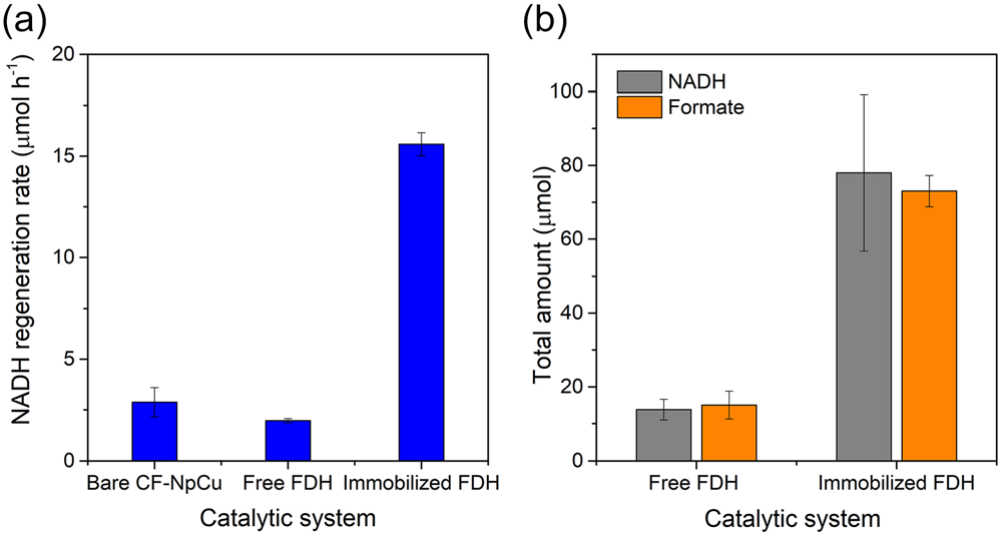
(a) Comparative NADH regeneration rates and (b) stoichiometric mass balance for the free and immobilized FDH systems after 5 h of CO_2_ conversion reaction (at −1.6 V vs. Ag/AgCl in 250 mM dipotassium phosphate buffer mM pH 8.5). In (a), the bare CF‐NpCu electrode serves as the control to establish the baseline electrochemical regeneration rate in the absence of the enzyme. Data are presented as the mean ± SD (*n* = 2).

To further validate the enzymatic activity of the electrochemically regenerated cofactor, a stoichiometric mass balance was performed over a 5‐h reaction period (Figure 6). A common challenge in bioelectrocatalysis is the nonspecific reduction of NAD^+^ which can lead to the formation of enzymatically inactive dimers (NAD_2_) or 1,6‐isomers [39]. To isolate the specific biocatalytic contribution, the total formate produced was corrected by subtracting the 0.6 mM background concentration measured with the control experiments (without FDH), which represents the direct (non‐enzymatic) CO_2_ reduction by the Cu nanoparticles.

As illustrated in Figure 6a, the immobilization of FDH exerts a profound synergistic effect on the system's kinetics. While the bare CF‐NpCu electrode and the soluble enzyme system exhibited similarly NADH regeneration rates (approximately 3 µmol h^−1^), the immobilized bioelectrode increased this rate five‐fold. This suggests that the proximity of the enzyme to the electrode surface facilitates a rapid interfacial turnover, effectively acting as a kinetic sink that pulls the reaction forward by the immediate consumption of the regenerated cofactor.

Consequently, the immobilized system demonstrates a high degree of stoichiometric efficiency (Figure 6b). The net enzymatic formate production (73 µmol) accounts for approximately 93% of the total NADH regenerated (78 µmol, calculated from the specific rates in Figure 6a). This high utilization rate provides definitive evidence that the CF‐NpCu electrode selectively yields the biologically active 1,4‐NADH isomer.

Beyond these laboratory‐scale metrics, the architectural design of the CF‐NpCu‐FDH system is inherently structured for industrial scale‐up. The scalability of the proposed CF‐NpCu‐FDH system is anchored in its material selection and architectural design. The use of 3D carbon felt as a scaffold provides a robust mechanical framework that is already a staple in industrial electrochemical technologies, such as vanadium redox flow batteries. Furthermore, the use of spray deposition for Cu nanoparticle modification offers a high‐throughput, commercially viable coating method that ensures reproducibility over large surface areas without the need for complex vacuum‐based infrastructure. When coupled with the straightforward affinity‐based immobilization protocols, these design choices facilitate a seamless transition from batch‐mode prototypes to continuous flow‐cell reactors (TRL 4 and beyond). While the current study operates in a batch configuration, the high stoichiometric efficiency (93%) and the stability of the bio‐interface under high cathodic stress (–1.6 V) provide the necessary experimental groundwork for transitioning to continuous flow‐cell reactors (TRL 4 and beyond). Consequently, the stability demonstrated at –1.6 V and the near‐stoichiometric utilization of the cofactor provide the necessary experimental groundwork to transition from this batch‐mode proof‐of‐concept toward continuous flow‐cell reactors (TRL 4 and beyond). Future efforts will focus on optimizing volumetric productivity in pressurized systems, further bridging the gap between fundamental laboratory findings and industrial pilot‐scale application.

## 4 Conclusions

This study demonstrates a highly efficient bioelectrocatalytic platform for CO_2_ conversion, centered on the strategic integration of NAD‐dependent FDH with nanoparticle‐modified 3D carbon felt. A key finding is the material‐specific synergy between the electrode and the cofactor: while SnO_2_, introduced here as an alternative cofactor‐regeneration catalyst, achieved the highest FE (10.5%) at lower potentials, Cu nanoparticles was identified as a superior kinetic driver for NADH regeneration, achieving the highest conversion (8.2%) and facilitating more effective redox coupling. This rate–selectivity trade‐off positions Cu as the optimal choice for high‐throughput bio‐interfaces, while SnO_2_ remains a promising, sustainable alternative that warrants further defect‐engineering to enhance its activity.

The development of the CF‐NpCu‐FDH bioelectrode, featuring a protein loading of 12 mg g^−1^, represents a significant advancement in bio‐interface design. By utilizing an affinity‐based immobilization strategy,  enzyme turnover was preserved while creating a ’biocatalytic driver’ effect; the immediate consumption of 1,4‐NADH at the interface acted as a kinetic sink, accelerating the regeneration rate fivefold compared with bare electrodes. This synergy resulted in a threefold increase in formate production over free‐enzyme systems, reaching a productivity of 43 µmol h^−1^ cm^−2^ with an exceptional stoichiometric efficiency of 93%.

Crucially, the scalability of this architecture is anchored in its industrial‐ready design. The use of 3D carbon felt—a staple in large‐scale flow battery technology—combined with straightforward nanoparticle deposition and robust NAD‐dependent enzymes, provides the experimental groundwork to transition from this batch‐mode proof‐of‐concept toward continuous flow‐cell reactors (TRL 4 and beyond). Future work will focus on implementing pH‐control systems and pressurized configurations to further enhance volumetric productivity, bridging the gap between fundamental laboratory findings and sustainable industrial CO_2_ upcycling.

## Supporting Information

Additional supporting information can be found online in the Supporting Information section.

## Author Contributions

**Diego Maureira**: investigation (lead), writing – original draft (lead). **Lorena Wilson**: conceptualization (supporting), formal analysis (equal), funding acquisition (supporting), supervision (supporting), writing – review and editing (equal). **Hilmar Guzmán**: data curation (supporting), formal analysis (supporting), investigation (supporting), methodology (equal), supervision (supporting), writing – review and editing (equal). **Tonia Tommasi**: data curation (supporting), formal analysis (supporting), writing review and editing (supporting). **Debora Fino**: funding acquisition (supporting), supervision: (supporting), writing review and editing (supporting). **Simelys Hernández**: conceptualization (supporting), data curation (supporting), formal analysis (supporting), methodology (lead), supervision (supporting), writing – review and editing (equal). **Carminna Ottone**: conceptualization (lead), data curation (lead), funding acquisition (lead), project administration (lead), supervision (lead), writing – review and editing (equal).

## Funding

This study was supported by the Agencia Nacional de Investigación y Desarrollo (ATE220045, 21190467) and the Erasmus+.

## Conflicts of Interest

The authors declare no conflicts of interest.

## Supporting information

Supplementary Material

## Data Availability

The data that support the findings of this study are available from the corresponding author upon reasonable request.
