# Supplementary material for: Cu and SnO2‐Modified Carbon Felt for Electroenzymatic CO2 Upcycling
Source: ChemSusChem. 2026 Apr 24;19(8):e202502316. doi: 10.1002/cssc.202502316 (PMC13109675; doi:10.1002/cssc.202502316)
Supplement: Supplementary file 1 — Supplementary Material [file CSSC-19-e202502316-s001.pdf]

Supporting Information

Cu and SnO_2-Modified Carbon Felt for Electroenzymatic CO_2 Upcycling

Diego Maureira[a], Lorena Wilson[a], Hilmar Guzmán[b], Tonia Tommasi[b], Debora Fino[b], Simelys Hernández[b]** and Carminna Ottone[a]*

[a] Escuela de Ingeniería Bioquímica
Pontificia Universidad Católica de Valparaíso (PUCV)
Av. Brasil 2085, Valparaíso, Chile.
E-mail: carminna.ottone@pucv.cl

[b] Department of Applied Science and Technology
Politecnico di Torino (PoliTO)
Corso Duca Degli Abruzzi 24, 10129, Turin, Italy
E-mail: simelys.hernandez@polito.it

*corresponding author: carminna.ottone@pucv.cl

**corresponding author: simelys.hernandez@polito.it

Field Code Changed

Contents

S1. Results obtained with bare CF electrode. 2
S2. Electrochemical NADH regeneration with CF-NpSnO_2 and CF-NpCu electrodes. 3
S3. Effect of using a high enzyme concentration. 4
S4. Control reaction without enzyme. 5
S5. Thermal stability 6

S1. Results obtained with bare CF electrode.

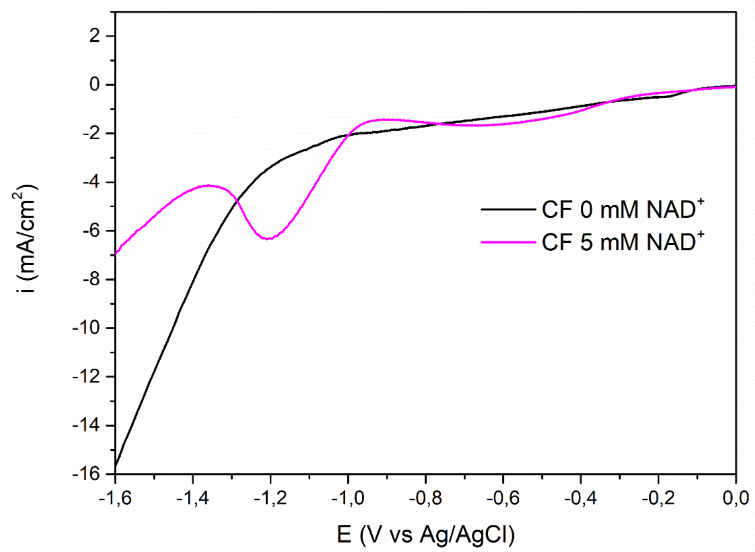

Fig. S1. Linear sweep voltammetry (LSV) of bare carbon felt (CF) in the absence (0 mM) and presence (5 mM) of NAD^+, performed at ambient temperature and pressure in 100 mM dipotassium phosphate buffer (pH 7).

S2. Electrochemical NADH regeneration with CF-NpSnO₂ and CF-NpCu electrodes.

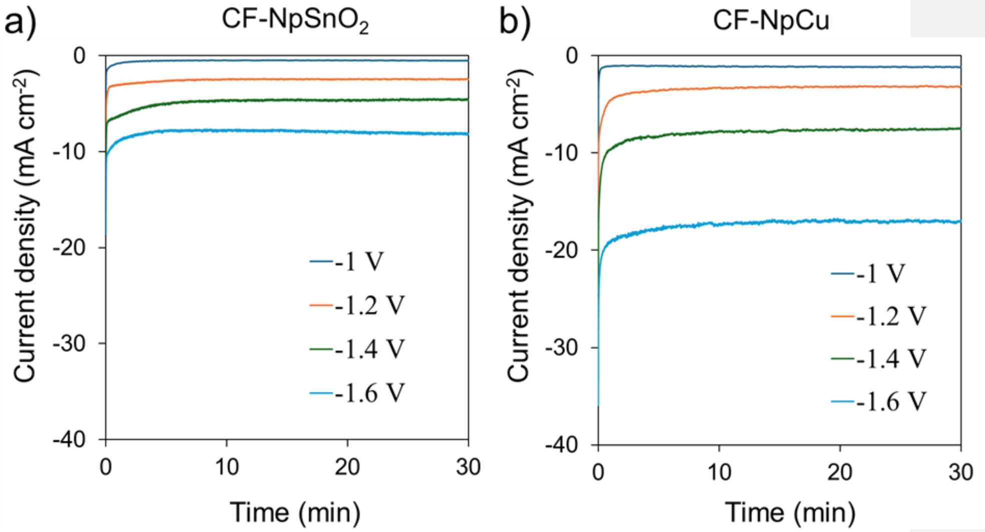

Figure S2. Chronoamperometry curves for electrochemical NADH regeneration using a) CF-NpCu and b) CF-NpSnO$_2$ electrodes at various applied potentials (–1.0, –1.2, –1.4, and –1.6 V vs Ag/AgCl) in a solution of 1 mM NAD$^+$ prepared in 100 mM dipotassium phosphate buffer (pH 7.0).

S3. Effect of using a high enzyme concentration.

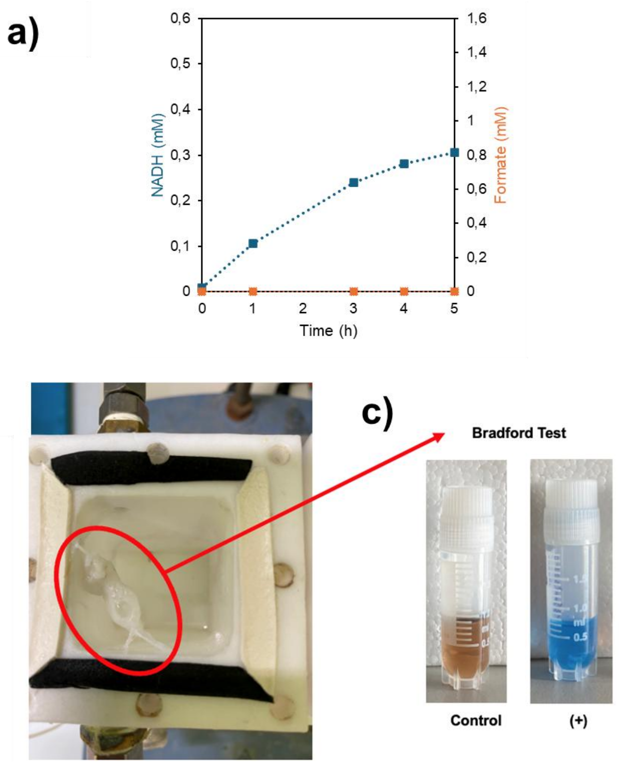

Fig. S3. Reaction performed at ambient temperature and pressure adding 1 U of free FDH in a 250 mM dipotassium phosphate buffer, pH 8.5 and 1mM NAD^+. A) Profile of formate and NADH during the reaction. B) Photograph of the reaction medium at the end of the electrochemical assay. C) Result of Bradford assay to the agglomerate of figure B and of a Control of only buffer. protein presence test using the Bradford reagent is shown, where the blue coloration indicates the presence of protein.

S4. Control reaction without enzyme.

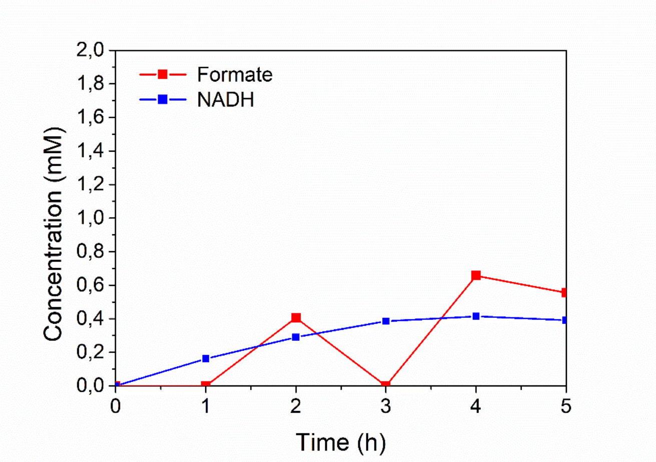

Fig S4. Profiles of formate and NADH production during electrochemical CO_2 conversion at –1.6 V vs Ag/AgCl using bare CF-NpCu electrode. Experiments were conducted at room temperature with a CO_2 flow rate of 30 NmL·min^{-1} in a solution of 1 mM NAD^+ prepared in 250 mM dipotassium phosphate buffer (pH 8.5).

S5. Thermal stability

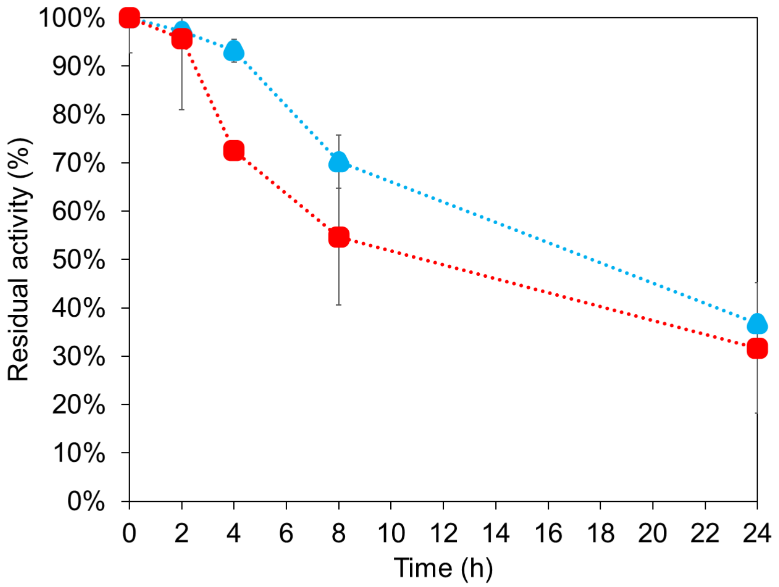

Fig. S5. Thermal stability assay under non-operational conditions with the bioelectrode (blue) and the free enzyme (red), incubated at 30 °C and pH 7.0.
